# Editorial: Cultural considerations in relation to mental health stigma

**DOI:** 10.3389/fpsyt.2024.1434319

**Published:** 2024-05-29

**Authors:** Mary Chambers, Maritta Välimäki, Petra C. Gronholm, Abdrabo Soliman

**Affiliations:** ^1^ Faculty of Health, Social Care and Education, Kingston University, London, United Kingdom; ^2^ School of Public Health, University of Helsinki, Helsinki, Finland; ^3^ Centre for Global Mental Health and Centre for Implementation Science, Health Service and Population Research Department, Institute of Psychiatry, Psychology & Neuroscience, King’s College London, London, United Kingdom; ^4^ Psychology Program, Department of Social Sciences, College of Arts and Sciences, Qatar University, Doha, Qatar

**Keywords:** stigma, discrimination, cultural influence, lived experience, mental health

Addressing the phenomenon of mental health stigma and discrimination necessitates a comprehensive understanding of the multifarious factors influencing individuals’ perceptions of health and wellbeing. Variability in cultural and societal perspectives on illness molds how individuals comprehend and respond to mental health concerns. This variability, shaped norms, beliefs, and values, critically influences attitudes and behavioral responses toward people experiencing mental health challenges. Stigma can hinder life opportunities, meaningful societal involvement, and the journey to healing, impacting not only those affected but also their families and broader social circles.

The recognition and integration of cultural considerations into the frameworks of strategies for understanding and reducing mental health stigma are imperative. Through this lens we can develop more effective ways to mitigate the negative impacts of stigma and discrimination, supporting social inclusion and building effective healthcare systems that cater to everyone.

To achieve such systems, it is important to actively involve patients/service users and carers (public contributors) in the commissioning and development of services, and in curriculum development of health and social care professionals. The active involvement of patients/service users and carers throughout the research process helps to ensure that outcomes address patient needs, are relevant to diverse communities, impact practice, facilitate sustainability, disseminate findings across patient/carer groups, and reduce stigma.

This editorial introduces an assembly of pivotal studies that illuminate the intricate nature of mental health stigma, underscored by a pronounced focus on the indispensable role of cultural considerations in understanding and addressing this concern. The editors’ collective endeavor in curating this compilation mirrors our profound commitment to nurturing a discourse on mental health stigma that is deeply rooted in cultural awareness.

At the forefront of this compilation is a seminal investigation by Daniel et al., which scrutinizes the cultural adaptation of INDIGO mental health stigma reduction interventions in North India. Through the application of the Ecological Validity Model (EVM), this study explored the cultural context needed to customize interventions that resonate with local cultural norms. This method not only amplifies the effectiveness of these interventions but also highlights the critical impact of cultural sensitivity within mental health initiatives. Strategic adaptations—addressing language, personal interactions, metaphors, content, methods, and context—exemplify the profound influence of deeply ingrained cultural elements in augmenting efforts to mitigate stigma.

Augmenting this perspective, Dambrun et al. delve into the social perceptions and stigmatization of mental illnesses in France, offering an insightful exploration into how the notions of vital force and burden contribute to social exclusion. This investigation, by providing the SUBAR model, not only sheds light on the unique facets of mental illness stigmatization within the French context but also highlights the complexity of stigma as a construct intricately woven with cultural narratives and societal frameworks.

Further augmenting this collection is the work of BinDhim et al., who focused on the cultural adaptation and validation of the Mental Illness Associated Stigma Scale (MIAS) for Arabic-speaking populations in Saudi Arabia. This study highlights the necessity of adapting research tools to specific cultural contexts, facilitating accurate stigma measurement.

In Tunisia, Ben Amor et al. validated the Arabic versions of the Mental Health Knowledge Schedule (MAKS) and the Reported and Intended Behavior Scale (RIBS) among Tunisian students. This endeavor not only confirms the psychometric soundness of these instruments in an Arabic-speaking milieu but also identifies sociodemographic and clinical determinants of mental illness stigma. This study explored a variety of factors that contribute to stigma, such as gender, academic background, personal history of health issues and previous encounters with illness. This set the stage for developing strategies to combat stigma effectively.

This compilation is further enriched by a study from van Beukering et al. examining Dutch workers’ attitudes toward colleagues with mental health conditions. The findings, which reveal diverse concerns and preferences regarding social distance, positions the workplace as a critical setting for stigma reduction efforts.

Li et al. embarked on an exploration of health stigma in China, concentrating on the unique experiences and challenges encountered by renal dialysis patients. Their study highlights the importance of developing interventions that consider the social backgrounds of the communities involved.

Similarly, Mpango et al. illuminated the physical and sexual victimization of individuals with severe mental illness in Uganda, highlighting the profound impact of stigma on vulnerable groups. This investigation emphasizes the necessity of formulating strategies aimed at preventing victimization and supporting survivors within culturally informed frameworks.

Odukoya et al. presented a comparative analysis on the efficacy of an e-intervention designed to diminish intellectual disability stigma among Nigerian and Kenyan internet users. This novel approach showcases the potential of digital platforms to overcome geographical and cultural barriers, paving new pathways for stigma reduction initiatives.

Finally, Scerri et al. offered a sociocultural examination of mental health stigma within the Maltese context, investigating the influence of cultural beliefs and societal norms on mental health perceptions and approaches. Their study emphasized the importance of family assistance, community unity and societal perceptions in influencing the journeys of individuals dealing with health struggles, emphasizing the necessity of culturally and contextually adapted interventions.

Collectively, the studies in this compilation not only shed light on the diverse manifestations of mental health stigma but also highlight the paramount importance of cultural sensitivity in addressing this pervasive issue. They advocate for an approach to mental health stigma that is deeply attuned to cultural contexts, calling for interventions that are both grounded in empirical evidence and aligned with cultural and contextual nuances.

In conclusion, this editorial and the accompanying suite of studies issue a compelling appeal for a culturally informed strategy for addressing mental health stigma. As the field progresses, it is imperative to leverage the insights from these studies, extending the boundaries of our understanding and interventions to foster an inclusive and empathetic global mental health landscape enabled via the ‘voice’ of service users/patients and carers. By undertaking this mission, we affirm our shared responsibility to tackle mental health stigma and discrimination, ensuring that cultural considerations remain at the heart of our efforts.

## Author contributions

MC: Writing – review & editing, Conceptualization. MV: Writing – review & editing, Conceptualization. PG: Writing – review & editing, Conceptualization. AS: Writing – review & editing, Writing – original draft, Conceptualization.

